# Mechanisims of asthma and allergic disease – 1069. Down regulation of IL-13 secretion in mononuclear cells by a beta-adrenergic blocker

**DOI:** 10.1186/1939-4551-6-S1-P66

**Published:** 2013-04-23

**Authors:** Fatemeh Hajighasemi

**Affiliations:** 1 Immunology, Department of Immunology, Faculty of Medicine, Shahed University, Tehran, Iran

## Background

Beta-adrenergic blockers such as propranolol have been commonly used for treatment of several cardiovascular complications such as arterial hypertension and arrhythmias. Anti-inflammatory effects of propranolol have also been reported. Interleukin-13 (IL-13) (a Th2-type cytokine) is a mediator of airway inflammation and increases in immediate-type allergy. In this study the effect of propranolol on IL-13 secretion in human peripheral blood mononuclear cells (hPBMCs) have been investigated in vitro.

## Methods

HPBMCs were used in this study. The cells were cultured in complete RPMI medium and then incubated with different concentrations of propranolol (4 ×10^-7^ - 4 ×10^-4^ M) for 24 hours. The level of IL-13 secreted in the cell culture supernatants was measured with the enzyme-linked immunosorbent assay (ELISA) kits (R&D systems).

## Results

Propranolol significantly and dose-dependently reduced IL-13 production in hPBMCs, compared to untreated control cells.

## Conclusions

According to the results of this study, propranolol considerably decreased the IL-13 expression in hPBMCs. Propranolol with its inhibitory effect on IL-13 production may be useful in alleviating the IL-13- induced respiratory inflammation in related diseases such as chronic obstructive pulmonary disease (COPD) and asthma. Therefore propranolol along with its chronic long-term usage in cardiac problems, might have potential implication in inflammatory disorders.

